# Effectiveness and safety of vedolizumab induction with or without budesonide in patients with moderately to severely active Crohn’s disease in Europe: a retrospective observational study

**DOI:** 10.1186/s12876-023-03032-7

**Published:** 2023-11-29

**Authors:** Roni Weisshof, Stephan R. Vavricka, Lieven Pouillon, Fiona Braegger, Montserrat Roset, Nawal Bent-Ennakhil, Marc Ferrante

**Affiliations:** 1 Department of Gastroenterology, Rambam Health Care Campus, Haifa, Israel; 2 Department of Gastroenterology and Hepatology, University Hospital Zürich, Zürich, Switzerland; 3 Center for Gastroenterology and Hepatology AG, Zürich, Switzerland; 4 Department of Gastroenterology and Hepatology, Imelda GI Clinical Research Center, Imeldaziekenhuis Bonheiden, Belgium; 5 EUCAN Evidence Generation, Takeda Pharmaceuticals International AG, Glattpark-Opfikon, Zürich, Switzerland; 6 IQVIA, Real World Solutions, Barcelona, Spain; 7 Department of Gastroenterology and Hepatology, University Hospitals Leuven, KU Leuven, Leuven, Belgium

**Keywords:** Budesonide, Crohn’s disease, Moderately to severely active crohn’s disease, Vedolizumab

## Abstract

**Background:**

Vedolizumab (VDZ), a gut-selective anti-lymphocyte trafficking integrin antibody, is effective in treating patients with moderately to severely active Crohn’s disease (CD). In this study, we examined the real-world effectiveness and safety of induction therapy using VDZ alone or in combination with budesonide (VDZ + BUD) among patients with CD in Belgium, Israel, and Switzerland.

**Methods:**

This retrospective chart review analysis included adult patients with moderately to severely active CD who started induction treatment with VDZ or VDZ + BUD (January 2015 through January 2019). The primary objective of this study was to assess the effectiveness in terms of clinical remission of VDZ alone or VDZ + BUD using patient-reported outcomes (PRO) of abdominal pain (AP) and/or loose stool frequency (LSF) (PRO-2) at weeks 0, 2, 6, 10, and 14. Regression models were used to assess differences and associations between the treatment groups.

**Results:**

Overall, 123 patients were included (VDZ, n = 73; VDZ + BUD, n = 50). Clinical remission rates at week 14 were 71.4% (50/70) and 68.0% (34/50) with VDZ and VDZ + BUD, respectively. Mean percentage change in AP and LSF from baseline to week 14 was comparable between the groups. Median (95% confidence interval [CI]) time to clinical remission was 91 [70.0–98.0] and 95 [70.0–98.0] days, respectively. One patient in each group discontinued VDZ and 68.0% of patients in the VDZ + BUD group discontinued BUD before week 14. The rates of overall adverse events were similar between the groups (VDZ, 23.3%; VDZ + BUD, 26.0%).

**Conclusions:**

In this retrospective study, VDZ alone and VDZ + BUD showed similar high remission rates in patients with moderately to severely active CD. Prospective randomized studies are needed to conclude on the role of combining VDZ with BUD.

**Trial registration:**

Not applicable.

**Supplementary Information:**

The online version contains supplementary material available at 10.1186/s12876-023-03032-7.

## Background

Crohn’s disease (CD) and ulcerative colitis, commonly referred to as inflammatory bowel diseases (IBD), are inflammatory diseases of the gastrointestinal tract and may develop due to complex interactions of inadequate immunological responses, genetic susceptibility, and environmental triggers [1]. Pharmacological treatment for CD involves a wide range of therapeutic agents with varying mechanisms of action including conventional (e.g., corticosteroids and immunomodulators) or advanced therapies (e.g., monoclonal antibodies and small molecules) [2, 3]. An update on the Selecting Therapeutic Targets in IBD (STRIDE-II) initiative of the International Organization for the Study of IBD recommends achieving a clinical response as an immediate treatment target for patients with IBD [4]. In addition to defining the appropriate medical treatment according to disease site and activity, the choice of medication for CD treatment is influenced by factors such as the balance between drug efficacy and potential side effects, previous response to treatment (in case of a relapse, or for a steroid-dependent or steroid-refractory disease), the presence of extraintestinal manifestations or complications, as well as the costs and benefit/risk ratio of each drug [3].

In clinical practice, induction of advanced therapies (e.g., monoclonal antibodies) such as vedolizumab (VDZ) with corticosteroids may improve clinical response and remission rates in patients with CD [5, 6]. VDZ is a gut-selective anti-lymphocyte trafficking integrin antibody with an attractive safety profile that is recommended for inducing response and remission in patients with moderately to severely active CD who have an inadequate response to conventional therapy and/or to anti–tumor necrosis factor (TNF) agents, or for maintaining clinical remission in patients who have achieved clinical remission with VDZ [3, 7–9]. Corticosteroids such as budesonide (BUD) have been recommended for inducing clinical remission in patients with active mild-to-moderate CD [3, 10–14]. BUD is a second-generation corticosteroid recommended for mild-to-moderate CD located at the ileum and/or ascending colon [3]. It has been shown to have high topical anti-inflammatory activity, fewer glucocorticoid-associated side effects, and less suppression of pituitary–adrenal function compared with systemic corticosteroids [10–14].

Clinical trials have demonstrated that VDZ treatment was more effective at 10 weeks than 6 weeks with respect to clinical remission, indicating that the benefits of VDZ treatment appear later in the treatment pathway [7]. As opposed to a systemic steroid, combining VDZ with a corticosteroid, such as BUD, which has a selective localized effect and a high first-pass metabolism in the liver, may expedite the time to clinical response and remission without jeopardizing safety and tolerability [5, 6, 10–14]. However, to date, there is limited clinical trial evidence for the use of VDZ + BUD and clinical guidelines do not have any recommendations for this treatment combination in patients with CD [3]. Therefore, it is pertinent to understand the effectiveness of VDZ + BUD, and to understand the factors that contribute to clinician prescribing decisions in real-world practice. The aim of this study was to assess the real-world effectiveness and safety after induction therapy using VDZ as monotherapy (VDZ alone) or VDZ + BUD in patients with moderately to severely active CD, along with the factors associated with the decision to prescribe VDZ + BUD.

## Methods

### Study design

**Fig. 1:**
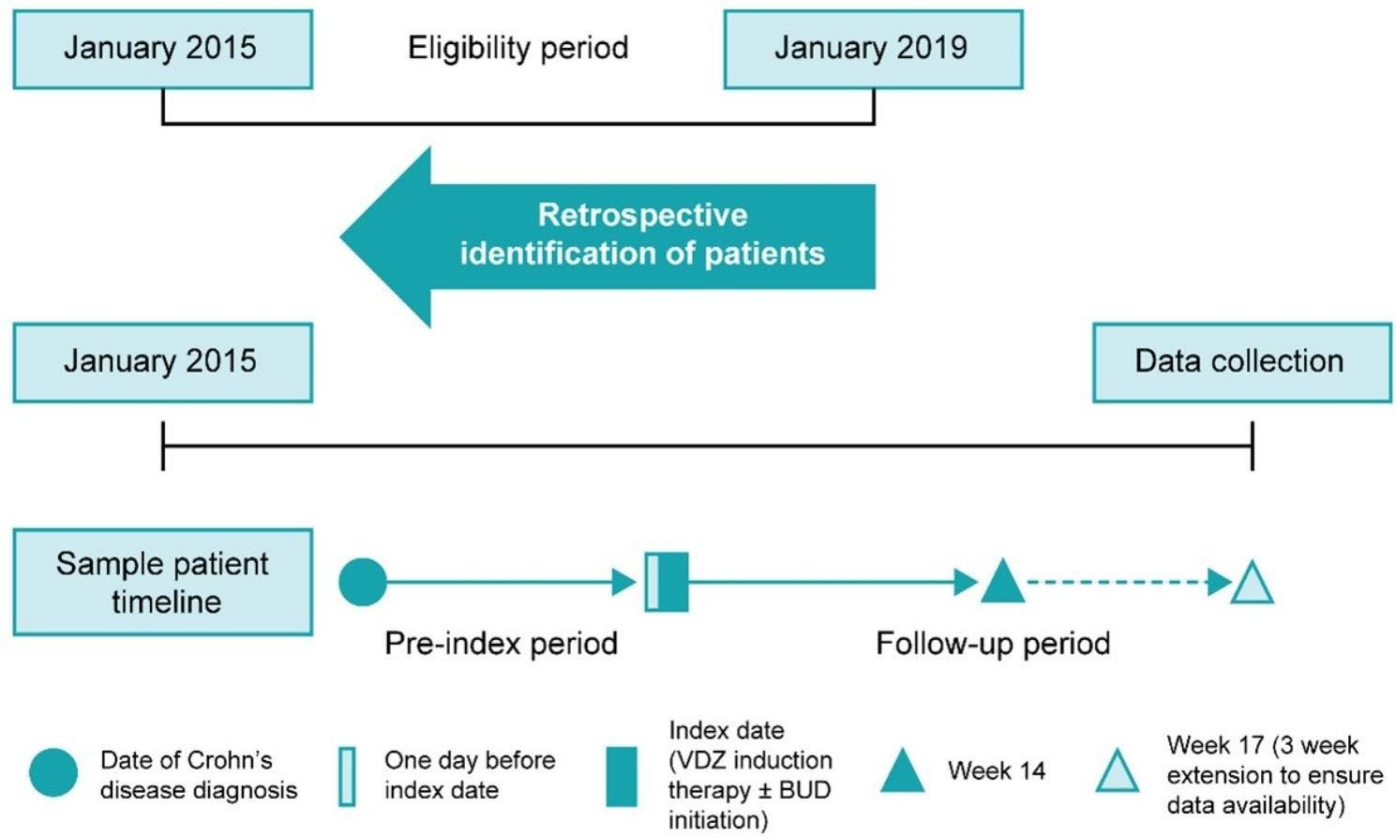
Study design. VDZ = vedolizumab; BUD = budesonide

This was a retrospective, multinational, multicenter medical chart review study of patients with moderately to severely active CD who initiated induction therapy with VDZ alone or VDZ + BUD (at least one week of BUD) between January 1, 2015, and January 31, 2019 (Fig. 1). The study was conducted at 11 centers in Belgium, Israel, and Switzerland. The index date was defined as the date of VDZ induction therapy with or without BUD initiation. The pre-index period began on the date of diagnosis of CD and ended 1 day before the index date. The post‐index period began 1 day after the index date and ended 14 weeks (+ 3 weeks extension to ensure data availability) after the index date, death, or loss to follow‐up, whichever occurred first. The follow-up period included the index date and the post-index period. This study was conducted in accordance with the Declaration of Helsinki and its amendments, International Conference on Harmonisation – Good Clinical Practice E6 guidelines, Good Pharmacoepidemiology Practices, the International Society for Pharmacoepidemiology’s Guidelines for Good Pharmacoepidemiology Practices, and any local regulations. Written informed consent or assent, as applicable, was obtained for data collection.

### Study population

The patient selection process is outlined in Additional file 1. Patients were included if they were aged ≥ 18 years, were diagnosed with moderately to severely active CD, had initiated induction therapy with VDZ during the study eligibility period (i.e., between January 1, 2015, and January 31, 2019), and had received either VDZ alone or VDZ + BUD at any dose of BUD at week 0 for a minimum duration of 1 week. To be eligible for inclusion, patients were required to have data for a minimum follow-up period of 14 weeks, including data on abdominal pain (AP) and/or loose stool frequency (LSF) within 1 week before induction and between weeks 12 and 14 (+ 3) of induction or at the time of treatment change. Patients were required to have active disease at the time of VDZ initiation, that is, at least moderate AP (≥ 2) and/or mean daily LSF ≥ 4 within 1 week before the index date. Patients were excluded if they were diagnosed with an indeterminate/unspecified type of IBD; were previously treated with VDZ; received any therapy for CD other than VDZ, BUD, or aminosalicylic acid at the index date (including systemic steroids); or were initiating induction therapy with VDZ as part of an interventional clinical trial. No standardization of BUD dosing protocols was used.

### Data collection

Data were extracted from patient medical charts into a web-based data entry tool; data were collected during the pre- and post-index periods. The collected data were stored in a secure data server located in France. To ensure patient anonymity, only server administrators were granted access to the server and its components. Patients were identified in the database only by their study ID, site ID, patient number, birth year, and sex. Sites consecutively selected the patient charts to begin data abstraction, starting with the most recent index date and working in reverse order towards the oldest index date. Coding for medical history, concomitant illness (defined using the Medical Dictionary of Regulatory Activities [MedDRA]), concomitant medication (World Health Organization Drug Dictionary), and adverse events (AEs)/reactions (MedDRA) was performed according to current standard coding instructions.

### Study outcomes

The primary objective of this study was to evaluate the effectiveness in terms of clinical remission of VDZ alone or VDZ + BUD using patient-reported symptoms of AP and/or LSF (PRO-2) during the follow-up period in patients with moderately to severely active CD. Clinical remission was defined based on an average daily composite score of the weighted total of patient-reported symptoms of AP (≤ 1) and LSF (≤ 3) [15, 16]. AP was assessed at baseline and at week 14 using the Likert scale (0 indicating no AP; 1, mild; 2, moderate; and 3, severe). A feasibility assessment of participant sites was performed to assess the frequency of AP score (APS) reporting in clinical charts at certain timepoints; this information formed the basis of the protocol to ensure suitable patient numbers. Information on the number of LSFs was collected at baseline and week 14. Mean percentage change from baseline to week 14 in patient-reported APS was calculated using the following formula:

$$\eqalign{ Mean\,percentage\,change\,in\,APS{\text{ }} = & {\text{ }}\left( {difference\,between\,final\,and\,baseline\,scores} \right)/ \cr & \left( {maximum{\text{ }}change{\text{ }}that{\text{ }}can{\text{ }}be{\text{ }}detected{\text{ }}in{\text{ }}the{\text{ }}variable{\text{ }} = {\text{ }}3} \right) \cr}$$

Mean percentage change in LSF from baseline to week 14 was calculated according to the following formula:

$$\eqalign{ Mean{\text{ }}percentage{\text{ }}change{\text{ }}in{\text{ }}LSF{\text{ }} = & {\text{ }}\left( {difference{\text{ }}in{\text{ }}LSF{\text{ }}between{\text{ }}final{\text{ }}and{\text{ }}baseline} \right)/ \cr & \left( {number{\text{ }}of{\text{ }}LSF{\text{ }}at{\text{ }}baseline} \right) \cr}$$

The secondary objectives were to evaluate [1] change in APS and/or LSF from baseline to weeks 2, 6, and 10 (or closest available before these timepoints); [2] time to clinical remission based on an average daily composite score of the weighted total of patient-reported symptoms of AP (≤ 1) and LSF (≤ 3); [3] incidence and characteristics of AEs. AEs were described using MedDRA and classified based on respective system organ classes (SOCs); [4] time to BUD withdrawal and reasons for withdrawal through week 14; [5] time to VDZ withdrawal and reasons for withdrawal through week 14; and [6] to describe the clinical, biochemical, and endoscopic assessment outcomes and previous treatment patterns. Exploratory objectives were [1] to evaluate changes in laboratory assessment measures (liver enzymes, hemoglobin, fecal calprotectin [fCal], and C-reactive protein [CRP] levels) and endoscopic findings through week 14 (or closest available before this timepoint); [2] to describe the safety profile depending on the regimen of VDZ administered during induction therapy; and [3] to identify any factors associated with a certain patient profile among patients who received VDZ alone vs. VDZ + BUD.

### Statistical analysis

Owing to the limited number of publications reporting data on the effectiveness of VDZ with and without steroids, such as BUD, assessed as the difference in change in AP and/or LSF, the sample size was calculated based on available outcomes. Due to the lack of published data at week 14, prior results of clinical remission at week 10 were considered for sample size calculation. The rate of clinical remission with VDZ at week 10 was estimated as 22.7%, and that with VDZ in combination with steroids was estimated as 34.2%. Based on these estimates, a minimum sample size of 68 and 87 patients in the VDZ alone and VDZ + BUD groups, respectively, was required to estimate the rate of clinical remission with a precision level of 0.10 (corresponding to a 95% confidence interval [CI] of ± 10%). A final sample size of 73 patients in the VDZ alone group and 50 patients in the VDZ + BUD group allowed for clinical remission to be estimated at precision levels below 0.10 and 0.13, respectively.

Descriptive statistics were used to summarize patient characteristics, clinical disease presentations, therapeutic regimens, and clinical outcomes. Continuous variables were presented as mean and standard deviation (SD) or as median and interquartile range (IQR). Categorical variables were presented as counts and proportions. Univariate and multivariate logistic regression models were used to identify the main factors associated with the decision to prescribe VDZ alone vs. VDZ + BUD. Variables included in the logistic regression models were defined based on data collected in the study (availability in clinical charts). All independent variables with a significance level of ≤ 0.1 in the univariate analysis comparing baseline characteristics between both treatment groups were included in the multivariate model as potential confounders. Mean percentage change in AP and/or LSF from baseline to week 14 (+ 3) was calculated for each treatment group. Kaplan-Meier curves were used for descriptive time-to-event analyses (time to BUD or VDZ discontinuation and time to clinical remission). The analysis was conducted by IQVIA (Barcelona, Spain) using SAS^®^ Enterprise Guide 7.13. The all patients enrolled (ENR) set was used for analysis and comprised all patients who provided informed consent and who fulfilled the selection criteria for this study.

## Results

### Patient disposition and baseline characteristics

A total of 123 patients, 73 in the VDZ alone group and 50 patients in the VDZ + BUD group, were included in the ENR analysis; however, not all patients were included for the various analysis sets. The demographic and disease characteristics at index date are outlined in Table 1 and Additional file 2. Most patients were female (57.7%), and the mean (SD) age was 44.2 (15.8) years. The most commonly reported disease location was L1 (solely terminal ileum; 51.9%), while the most seen disease behavior was B1 (non-stricturing, non-penetrating; 66.0%). Physician-rated disease activity at index date revealed that 74.0% and 16.3% of the patients had moderate and severe disease activity, respectively. A total of six patients (4.9%) in the overall study population did not receive any treatment for CD before the index date. The proportion of patients with no prior treatment was higher in the VDZ + BUD group (10.0%) than in the VDZ alone group (1.4%). The most common prior treatments were anti-TNF agents (83.3% and 88.9%), followed by immunomodulators (76.4% and 80.0%) and topical corticosteroids (63.9% and 64.4%) in the VDZ alone group and VDZ + BUD group, respectively. Chronic comorbidities were present in > 50% of patients in both groups and are shown in Additional file 2.

**Table 1:** Demographic and disease characteristics at index date

Characteristic	VDZ alone (N = 73)	VDZ + BUD (N = 50)	Overall population (N = 123)	P-value
**Age, years, median (IQR)**	45.0 (32.0, 56.0)	42.0 (30.0, 50.0)	45.0 (30.0, 55.0)	0.5494
**Female, n (%)**	39 (53.4)	32 (64.0)	71 (57.7)	0.2447
**Smoking status, n (%)**				0.9971
** Current**	23 (31.5)	16 (32.0)	39 (31.7)	
** Former**	12 (16.4)	8 (16.0)	20 (16.3)	
**Duration of CD**^**a**^, **n (%)**				0.0961
** < 2 years**	9 (12.3)	12 (24.0)	21 (17.1)	
** ≥ 2 years**	64 (87.7)	38 (76.0)	102 (82.9)	
**Physician assessment of disease activity, n (%)**				0.6537
** Not evaluated**	7 (9.6)	5 (10.0)	12 (9.8)	
** Moderate**	55 (75.3)	36 (72.0)	91 (74.0)	
** Severe**	11 (15.1)	9 (18.0)	20 (16.3)	
**Abdominal pain**^**b**^, **n (%)**				0.1436
** None**	7 (9.6)	1 (2.0)	8 (6.5)	
** Mild**	8 (11.0)	2 (4.0)	10 (8.1)	
** Moderate**	53 (72.6)	40 (80.0)	93 (75.6)	
** Severe**	5 (6.8)	7 (14.0)	12 (9.8)	
**Mean daily number ** **of loose stools**^**b**^, **median (IQR)**	5.0 (4.0, 7.0)	5.0 (4.0, 6.5)	5.0 (4.0, 7.0)	0.3948
**Fecal calprotectin**^**c**^, **n/N (%)**				NA
** ≤ 250 µg/g**	2/61 (3.3)	4/34 (11.8)	6/95 (6.3)	
** > 250 µg/g**	18/61 (29.5)	4/34 (11.8)	22/95 (23.2)	
**CRP**^**c**^, **n/N (%)**				NA
** < 0.8 mg/L**	2/61 (3.3)	5/34 (14.7)	7/95 (7.4)	
** ≥ 0.8 mg/L**	55/61 (90.2)	28/34 (82.4)	83/95 (87.4)	
***Prior treatment for CD***^**d, e**^, **n (%)**				
** No treatment**	1 (1.4)	5 (10.0)	6 (4.9)	
** Anti-TNF agents**	60 (83.3)	40 (88.9)	100 (85.5)	0.7594
** Immunomodulators**	55 (76.4)	36 (80.0)	91 (77.8)	0.6781
** Aminosalicylates**	26 (36.1)	12 (26.7)	38 (32.5)	0.1708
** Corticosteroids (topical)**	46 (63.9)	29 (64.4)	75 (64.1)	0.5756
** Corticosteroids (systemic)**	5 (6.9)	5 (11.1)	10 (8.5)	0.5300
**Disease location (Montreal classification), n (%)**				NA
** L1 ileum**	34 (54.8)	21 (47.7)	55 (51.9)	
** L2 colon**	12 (19.4)	6 (13.6)	18 (17.0)	
** L3 ileocolon**	13 (21.0)	15 (34.1)	28 (26.4)	
** L1 + L4 upper gastrointestinal tract**	2 (3.2)	1 (2.3)	3 (2.8)	
** L2 + L4 upper gastrointestinal tract**	0 (0.0)	1 (2.3)	1 (0.9)	
** L3 + L4 upper gastrointestinal tract**	1 (1.6)	0 (0.0)	1 (0.9)	

^a^Assessed since CD diagnosis until the index date

^b^Reported by the patient and not evaluated at index date

^c^Overall, 28 patients were not evaluated for laboratory assessments (including CRP, fecal calprotectin, hemoglobin, and serum albumin) at index date

^d^Assessed since CD diagnosis until index date; percentages calculated from the number of patients receiving prior treatments for CD. Treatments prescribed were grouped using the following categories: Anti-TNF agents: adalimumab, certolizumab pegol, golimumab, infliximab. Immunomodulators: azathioprine, mercaptopurine, methotrexate. Aminosalicylates: mesalazine, sulfasalazine. Corticosteroids (topical): budesonide (MMX), beclomethasone dipropionate. Corticosteroids (systemic): methylprednisolone, prednisone

^e^Two patients in the VDZ + BUD group received prior treatment with other immunosuppressant monoclonal antibodies (natalizumab and ustekinumab)

BUD = budesonide; CD = Crohn’s disease; CRP = C-reactive protein; IQR = interquartile range; NA = not available; TNF = tumor necrosis factor; VDZ = vedolizumab

### Clinical remission

**Fig. 2:**
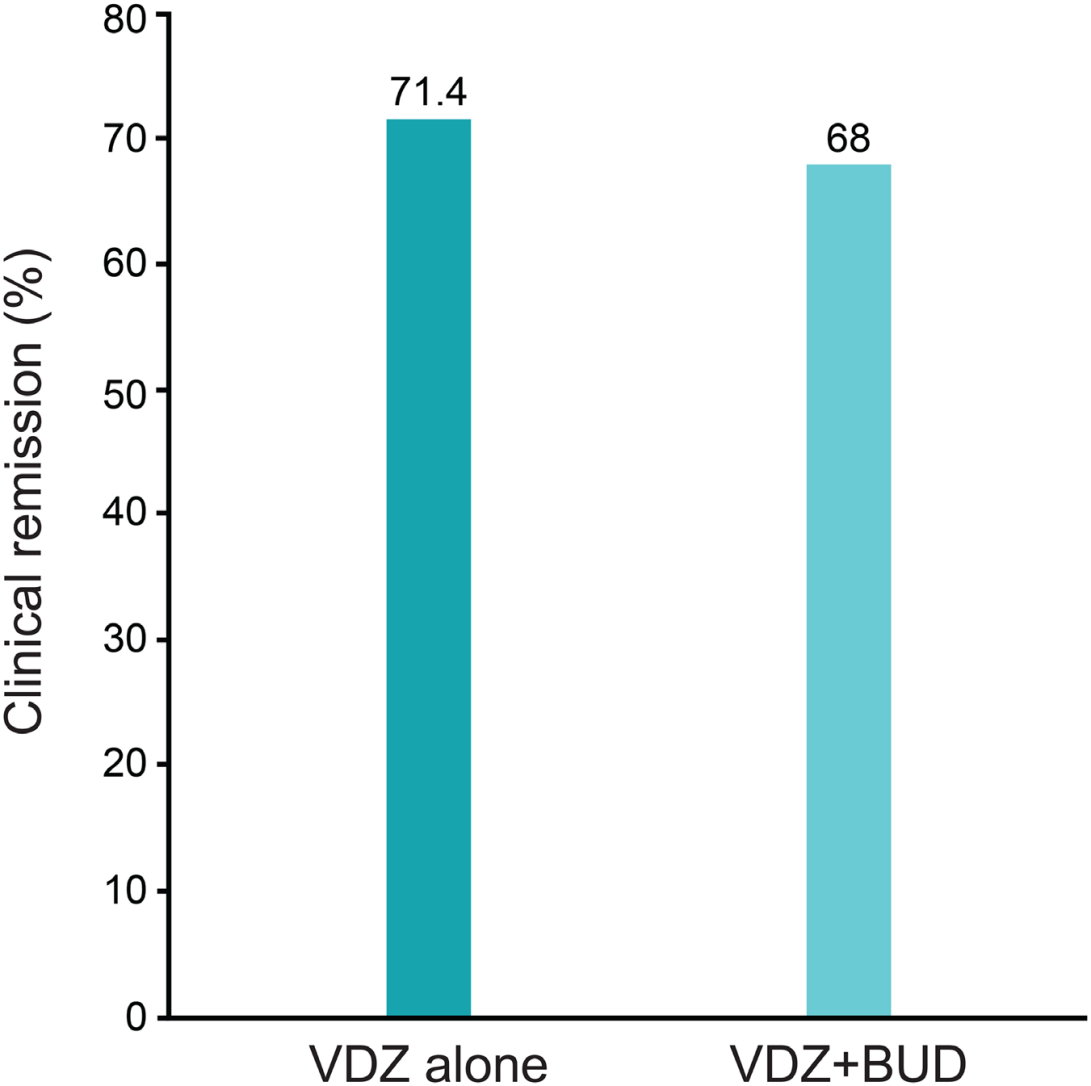
Clinical remission rates at week 14. VDZ = vedolizumab; BUD = budesonide

A total of 70/73 and 50/50 patients were included in the VDZ alone and VDZ + BUD groups, respectively, and clinical remission at week 14 was achieved in 71.4% and 68.0% of patients in the VDZ alone and VDZ + BUD groups, respectively (Fig. 2).

### Mean percentage change in APS and LSF from baseline to week 14

**Fig. 3:**
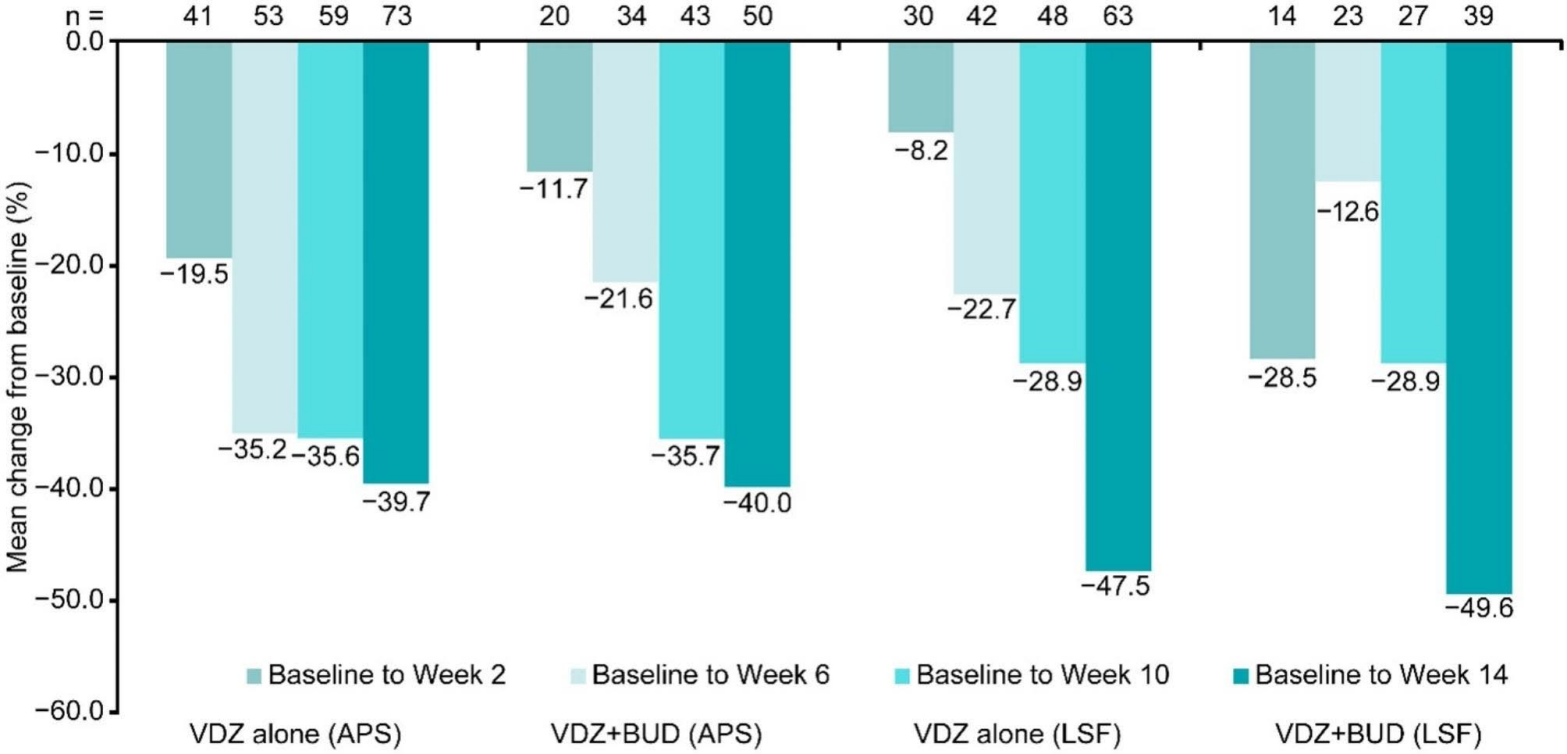
Mean percentage change in APS and LSF from baseline. APS = abdominal pain score; BUD = budesonide; LSF = loose stool frequency; VDZ = vedolizumab

The overall mean (SD) percentage change in APS from baseline to week 14 was − 39.8% (27.5%), and the change values were − 39.7% (29.2%) and − 40.0% (25.2%) for the VDZ alone and VDZ + BUD groups, respectively. The overall mean (SD) percentage change in LSF from baseline to week 14 was − 48.3% (69.1%), and the change values were − 47.5% (68.2%) and − 49.6% (71.4%) for the VDZ alone and VDZ + BUD groups, respectively (Fig. 3).

### Time to clinical remission

**Fig. 4:**
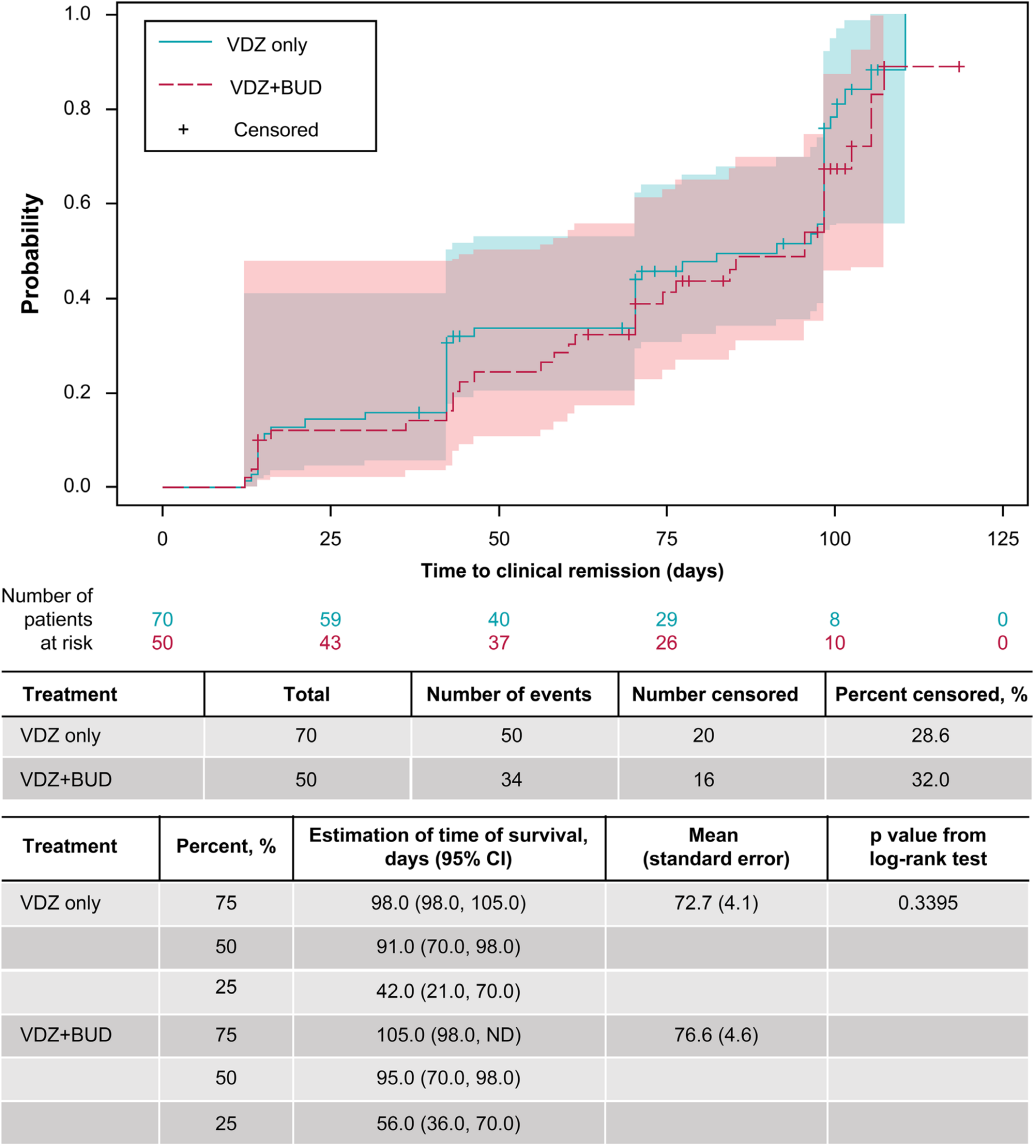
Time to clinical remission (days) according to patient-reported symptoms: APS (≤ 1) and LSF (≤ 3). PRO-2 is estimated as a sum of the weighted averages of APS and LSF. APS = abdominal pain score; BUD = budesonide; CI = confidence interval; LSF = loose stool frequency; VDZ = vedolizumab

Median time to clinical remission based on AP and LSF over time was 91 days (95% CI: 70.0, 98.0) and 95 days (95% CI: 70.0, 98.0), respectively. The percentage of censored patients was lower in the VDZ group (28.6%) than in the VDZ + BUD group (32.0%) (Fig. 4).

### Safety outcomes

Overall, 30 patients (24.4%) reported at least one AE over a period of 14 weeks. The most reported AEs per SOC were gastrointestinal disorders (40.0%), followed by infections and infestations (33.3%; Table 2). Overall, nine patients (7.3%) reported serious AEs (SAEs). Of the nine patients, two (22.2%) experienced at least one severe SAE, and seven (77.8%) completely recovered from SAEs. Analysis of AEs and SAEs in patients who received BUD at a dose of ≥ 9 mg/day showed that 12 patients (27.3%) reported AEs and three patients (6.8%) reported SAEs. Of the 12 patients having AEs, eight (66.7%) experienced a documented reaction to BUD. The dose of BUD was not changed in five patients (62.5%), was reduced or withdrawn in one patient (12.5%) each, while no action was taken in one patient.

**Table 2:** Safety outcomes

Parameters, n (%)		VDZ alone (N = 73)	VDZ + BUD (N = 50)	Overall population (N = 123)	p-value
**AE**		17 (23.3)	13 (26.0)	30 (24.4)	0.7308
**Severity of AE**	Mild	7 (41.2)	10 (76.9)	17 (56.7)	
	Moderate	8 (47.1)	7 (53.8)	15 (50.0)	
	Severe	3 (17.6)	1 (7.7)	4 (13.3)	
**SAE**		6 (8.2)	3 (6.0)	9 (7.3)	0.6424
**Severity of SAE**	Mild	0 (0.0)	0 (0.0)	0 (0.0)	-
	Moderate	5 (83.3)	2 (66.7)	7 (77.8)	
	Severe	1 (16.7)	1 (33.3)	2 (22.2)	
**Description of AE > 5%**	**GI disorders**	6 (35.3)	6 (46.2)	12 (40.0)	-
	Abdominal distension	1 (16.7)	1 (16.7)	2 (16.7)	
	Anal fissure	1 (16.7)	1 (16.7)	2 (16.7)	
	Bile acid malabsorption	0 (0.0)	1 (16.7)	1 (8.3)	
	Constipation	0 (0.0)	1 (16.7)	1 (8.3)	
	Worsening Crohn’s disease	3 (50.0)	0 (0.0)	3 (25.0)	
	Flatulence	1 (16.7)	1 (16.7)	2 (16.7)	
	Nausea	1 (16.7)	1 (16.7)	2 (16.7)	
	Toothache	1 (16.7)	0 (0.0)	1 (8.3)	
	Vomiting	0 (0.0)	1 (16.7)	1 (8.3)	
	**Infections and infestations**	6 (35.3)	4 (30.8)	10 (33.3)	
	Anal abscess	1 (16.7)	1 (25.0)	2 (20.0)	
	Cytomegalovirus infection	1 (16.7)	0 (0.0)	1 (10.0)	
	Gastroenteritis	1 (16.7)	1 (25.0)	2 (20.0)	
	Overgrowth bacterial	0 (0.0)	1 (25.0)	1 (10.0)	
	Pneumonia	1 (16.7)	0 (0.0)	1 (10.0)	
	Upper respiratory tract infection	2 (33.3)	0 (0.0)	2 (20.0)	
	Urinary tract infection	0 (0.0)	1 (25.0)	1 (10.0)	
	**Musculoskeletal and connective tissue disorders**	7 (41.2)	1 (7.7)	8 (26.7)	
	Arthralgia	4 (57.1)	0 (0.0)	4 (50.0)	
	Muscular weakness	0 (0.0)	1 (100.0)	1 (12.5)	
	Osteoarthritis	1 (14.3)	0 (0.0)	1 (12.5)	
	Pain in the extremity	2 (28.6)	0 (0.0)	2 (25.0)	
	**General disorders and administration site conditions**	3 (17.6)	5 (38.5)	8 (26.7)	
	Fatigue	2 (66.7)	4 (80.0)	6 (75.0)	
	Edema peripheral	0 (0.0)	1 (20.0)	1 (12.5)	
	Pyrexia	1 (33.3)	0 (0.0)	1 (12.5)	
	Rash	1 (33.3)	2 (40.0)	3 (37.5)	
	**Nervous system disorders**	2 (11.8)	5 (38.5)	7 (23.3)	
	Dizziness	0 (0.0)	1 (20.0)	1 (14.3)	
	Epilepsy	0 (0.0)	1 (20.0)	1 (14.3)	
	Headache	2 (100.0)	4 (80.0)	6 (85.7)	
	**Skin and subcutaneous tissue disorders**	2 (11.8)	1 (7.7)	3 (10.0)	
	Eczema	1 (50.0)	0 (0.0)	1 (33.3)	
	Erythema	1 (50.0)	0 (0.0)	1 (33.3)	
	Pruritus	0 (0.0)	1 (100.0)	1 (33.3)	
	**Surgical and medical procedures**	1 (5.9)	1 (7.7)	2 (6.7)	
	**Investigations**	0 (0.0)	2 (15.4)	2 (6.7)	
**Outcome related to AE**	Fatal	0 (0.0)	0 (0.0)	0 (0.0)	-
	Recovered/resolved	15 (88.2)	10 (76.9)	25 (83.3)	
	Recovering/resolving	1 (5.9)	5 (38.5)	6 (20.0)	
	Not recovered/not resolved	4 (23.5)	2 (15.4)	6 (20.0)	
	Recovered with sequelae/resolved with sequelae	0 (0.0)	0 (0.0)	0 (0.0)	
	Unknown	2 (11.8)	0 (0.0)	2 (6.7)	

AE = adverse event; BUD = budesonide; GI = gastrointestinal; SAE = serious adverse event; VDZ = vedolizumab

### Factors associated with the decision to prescribe VDZ + BUD

In a univariate analysis, the use of VDZ + BUD as an induction therapy was less common in patients who had received one prior biologic treatment (85.5% of the overall population had received prior biologic treatment) compared with patients who were biologic-naive (odds ratio [OR] = 0.40; 95% CI: 0.16, 0.98; p = 0.0455). Patients with a CD duration of ≥ 2 years were less often prescribed VDZ + BUD (OR = 0.45; 95% CI: 0.17, 1.16; p = 0.0961) (Table 3). Also, patients whose weight was in the range of 60–74 kg were prescribed VDZ + BUD less often (OR = 0.31; 95% CI: 0.11, 0.89; p = 0.0288) compared with patients with a lower body weight. In a multivariate analysis, the latter was the only difference between patients prescribed VDZ + BUD compared with VDZ alone (OR = 0.28; 95% CI: 0.09, 0.91; p = 0.0338). The result regarding the use of one prior biologic treatment compared with no prior biologic treatment was no longer statistically significant (OR = 0.39; 95% CI: 0.15, 1.03; p = 0.0570).

**Table 3:** Factors associated with the decision to prescribe VDZ + BUD

Univariate logistic regression model	Odds ratio (95% CI)		p-value (for category)	p-value (for variable)	
**Sex (ref = Male)**	Female	1.55 (0.74, 3.24)	-	0.2447	
**Age (ref = > 75 years old)**	< 30 years old	1.78 (0.15, 20.86)	0.6470		
	30–49 years old	0.97 (0.08, 11.57)	0.9779		
	50–75 years old	1.47 (0.12, 18.29)	0.7661		
**Weight (ref = < 60 kg)**	60–74 kg	0.31 (0.11, 0.89)	0.0288		
	> 74 kg	0.56 (0.20, 1.52)	0.2530		
**Smoking status (ref = Non-smoker)**	Former smoker	0.97 (0.35, 2.74)	0.9575		
	Current smoker	1.02 (0.45, 2.31)	0.9726		
**Site location (ref = Belgium)**	Switzerland	1.63 (0.63, 4.26)	0.3147		
	Israel	0.54 (0.16, 1.83)	0.3234		
**Charlson Comorbidity Index**		0.92 (0.71, 1.19)	-	0.5185	
**Number of prior biologics received (ref = 0)**	1	0.40 (0.16, 0.98)	0.0455		
	> 1	1.56 (0.55, 4.41)	0.4064		
**AP (ref = None)**	Mild	1.75 (0.13, 23.67)	0.6743		
	Moderate	5.28 (0.62, 44.61)	0.1266		
	Severe	9.79 (0.90, 106.68)	0.0612		
**LSF**	Number	0.95 (0.84, 1.07)	-	0.3948	
**AP/LSF score**	Number	0.98 (0.87, 1.10)	-	0.7062	
**Phenotype (ref = Non-stricturing, non-penetrating)**	Penetrating	0.41 (0.08, 2.11)	0.2858		
	Stricturing	1.43 (0.57, 3.62)	0.4461		
**Location of CD (ref = Ileum)**	Colonic	0.96 (0.33, 2.79)	0.9323		
	Ileocolonic	1.75 (0.71, 4.32)	0.2219		
**Duration of CD (ref = < 2 years)**	≥ 2 years	0.45 (0.17, 1.16)	-	0.0961	
**Prior CD-related surgery (ref = No)**	Yes	0.76 (0.37, 1.58)	-	0.4664	
***Multivariate regression model***					
**Weight (ref = < 60 kg)**	60–74 kg	0.28 (0.09–0.91)	0.0338		
	> 74 kg	0.63 (0.21–1.87)	0.4044		
**Number of prior biologics received (ref = 0)**	1	0.39 (0.15–1.03)	0.0570		
	> 1	1.01 (0.27–3.71)	0.9925		

AP = abdominal pain; BUD = budesonide; CD = Crohn’s disease; CI = confidence interval; LSF = loose stool frequency; ref = reference group; VDZ = vedolizumab

### Treatment regimen and discontinuation

A total of 27 (37.0%) and 14 (28.0%) patients in the VDZ alone and VDZ + BUD groups, respectively, received 300 mg VDZ by intravenous infusion at 0, 2, 6, and 14 weeks. A total of 19 (26.0%) and 21 (42.0%) patients received a supplemental dose of 300 mg VDZ at week 10 in the VDZ alone and VDZ + BUD groups, respectively. The VDZ regimen was not altered in 119 of the 123 patients (96.7%) during the follow-up period, and this was similar across the treatment groups. The reasons for treatment alteration in four patients were partial treatment response to therapies for managing CD (n = 3) and an unknown reason (n = 1). The VDZ treatment regimen was increased in dose (n = 1), increased in frequency (n = 2), or reduced in frequency (n = 1). VDZ treatment discontinuation prior to week 14 was reported in two patients, one patient in each group due to lack of effectiveness (VDZ alone) or occurrence of an AE (headache; VDZ + BUD). The median (range) BUD dose was 9.0 (3.0, 9.0) mg (Additional file 3). By the end of the follow-up period, 68.0% of patients in the VDZ + BUD group discontinued BUD. Reasons for BUD discontinuation included the classical tapering schedule for BUD (n = 29, 85.3%), lack of effectiveness for the management of CD (n = 2, 5.9%), and AEs (n = 1, 2.9%).

### Laboratory values and endoscopic findings at week 14

In the overall population, changes (median [IQR]) in serum albumin (g/dL), CRP (mg/L), and hemoglobin (g/dL) levels by week 14 were 2.2 (1.9, 2.4), 2.8 (–0.5, 8.0), and 11.2 (10.0, 12.1), respectively. The change in laboratory values were similar in both treatment groups. At the end of follow-up, lesions in the rectum and ileum were reported in one patient each of 73 patients (VDZ alone group). Endoscopic remission, defined as the absence of deep or superficial ulceration, was observed in one out of two patients at week 14.

## Discussion

This retrospective, real-world study assessed the effectiveness and safety of VDZ as an induction therapy with or without BUD in patients with moderately to severely active CD. Based on the PRO-2 parameters of APS and LSF according to the STRIDE-II recommendations, a high clinical remission rate (71.4% and 68.0% with VDZ alone and VDZ + BUD, respectively) at 14 weeks and similar median time to clinical remission were observed in both the treatment groups. Overall, the mean percentage change in APS was similar for the VDZ alone and VDZ + BUD groups from baseline to week 14, whereas the change in LSF was slightly lower in the VDZ alone group than in the VDZ + BUD group.

Studies with a 12-month follow-up period, such as the pivotal large-scale GEMINI 1 and VISIBLE 1 clinical trials, have reported clinical remission rates of approximately 42–46% [17, 18]. This real-world study demonstrated higher remission rates already at week 14 (71.4% and 68.0% with VDZ alone and VDZ + BUD, respectively), assessed using PRO-2.

This could potentially be explained by the exclusion of patients on systemic corticosteroids or selection bias due to the real-world retrospective nature of this study. The GEMINI 2 and GEMINI 3 sub-analysis outcomes indicated significant decreases in AP and LSF scores as early as week 4 [5]; the mean percentage change in APS and LSF was also high in this study. An exploratory sub-analysis of the GEMINI 2 and GEMINI 3 studies evaluated a subgroup of patients receiving VDZ in combination with continued stable corticosteroids (≤ 30 mg/day of prednisone or ≤ 9 mg/day BUD or equivalent dosing with another corticosteroid) for induction therapy in CD, and higher rates of clinical response and clinical remission were reported for patients receiving VDZ in combination with corticosteroids than for patients receiving VDZ alone during induction therapy [5]. In contrast, the remission rate observed in the current study was slightly lower for the VDZ + BUD group.

Patient profiles across the two study groups differed for some demographic and baseline characteristics, although the severity of CD was similar across both groups. The prescribing analysis suggested that patients’ body weight and prior use of anti-TNF agents were potential predictors of the choice between VDZ alone and VDZ + BUD groups; however, a robust clinical profile could not be inferred. Moreover, this study did not permit the identification of other clinical measures such as disease severity or location as potential drivers for the decision to prescribe VDZ + BUD.

This study adds to the existing evidence by exploring a “de novo” combination of two therapies with limited systemic immunosuppressive effects in comparison with other studies that have reported on subpopulations of patients who were on either systemic corticosteroids or other immunosuppressive therapies [5]. Additionally, data from this study allow for real-world treatment patterns to be observed outside the controlled environment of clinical trials, aiding in the understanding of how treatments are used in clinical practice; however, it should be noted that variables included in the logistic regression models were defined based on data availability in clinical charts only. Limitations of this study include the retrospective study design and the heterogeneity of data reporting as the original data were not intended for research. For instance, the duration and dosage of treatment with budesonide varied during follow-up, and 13.6% of patients in the VDZ + BUD group had L2 colonic disease (without further specification), while budesonide is recommended for CD located at the ileum and/or ascending colon [3]. Furthermore, the study was not designed or powered to conduct robust statistical comparisons between the treatment groups. The lack of identification of clinical drivers (i.e., disease location or severity) for the use of the VDZ + BUD and the predominant clinical characteristics of the patient sample (e.g., L1 disease location) limit generalizability. There is also potential for systematic under-recording of information in medical charts, which might have resulted in missing values. The inclusion criteria for data availability may also have introduced a selection bias for patients with complete medical charts, although the use of 14-week data availability as an inclusion criterion reduced the number of missing values for the primary objective. Endoscopic data were lacking, as disease evolution in CD is typically evaluated at months 6–9 after initiating a new therapy [19], and the follow-up period of the current study was shorter than this. Finally, there is a possibility that channeling bias could have been introduced because patients who received VDZ + BUD throughout the induction period had baseline characteristics different from those who received VDZ alone, thus affecting the overall outcome of the groups in terms of changes in both APS and LSF and remission rates.

## Conclusions

In this real-world study of patients with moderately to severely active CD, high rates of clinical remission were observed with VDZ alone and VDZ + BUD with a favorable safety profile, despite differences in patient characteristics between the groups. Although this study addresses an evidence gap relating to treatment with VDZ + BUD, only prospective and randomized controlled trials with larger populations can further evaluate the difference between VDZ alone and VDZ + BUD.

### Electronic supplementary material

Below is the link to the electronic supplementary material.

Supplementary Material 1

Supplementary Material 2

Supplementary Material 3

## Data Availability

The data sets generated and/or analyzed during the current study are available from the corresponding author on reasonable request to researchers who provide a methodologically sound proposal. The data will be provided after its de-identification in compliance with applicable privacy laws, data protection, and requirements for consent and anonymization.

## List of abbreviations

AP: Abdominal pain
APS: Abdominal pain score
AE: Adverse event
BUD: Budesonide
CI: Confidence interval
CRP: C-reactive protein
CD: Crohn’s disease
ECCO: European Crohn’s and Colitis Organisation
fCal: Fecal calprotectin
IBD: Inflammatory bowel disease
IQR: Interquartile range
LSF: Loose stool frequency
MedDRA: Medical Dictionary of Regulatory Activities
OR: Odds ratio
PRO: Patient-reported outcome
STRIDE-II: Selecting Therapeutic Targets in Inflammatory Bowel Disease
SAE: Serious adverse event
SD: Standard deviation
SOC: System organ class
TNF: Tumor necrosis factor
VDZ: Vedolizumab
