# Supplementary material for: Effectiveness and safety of vedolizumab induction with or without budesonide in patients with moderately to severely active Crohn’s disease in Europe: a retrospective observational study
Source: BMC Gastroenterol. 2023 Nov 29;23:417. doi: 10.1186/s12876-023-03032-7 (PMC10688148; doi:10.1186/s12876-023-03032-7)
Supplement: Supplementary file 1 — Supplementary Material 1 [file 12876_2023_3032_MOESM1_ESM.docx]

**Additional file 1.**

Supplementary Figure 1: Patient selection flowchart


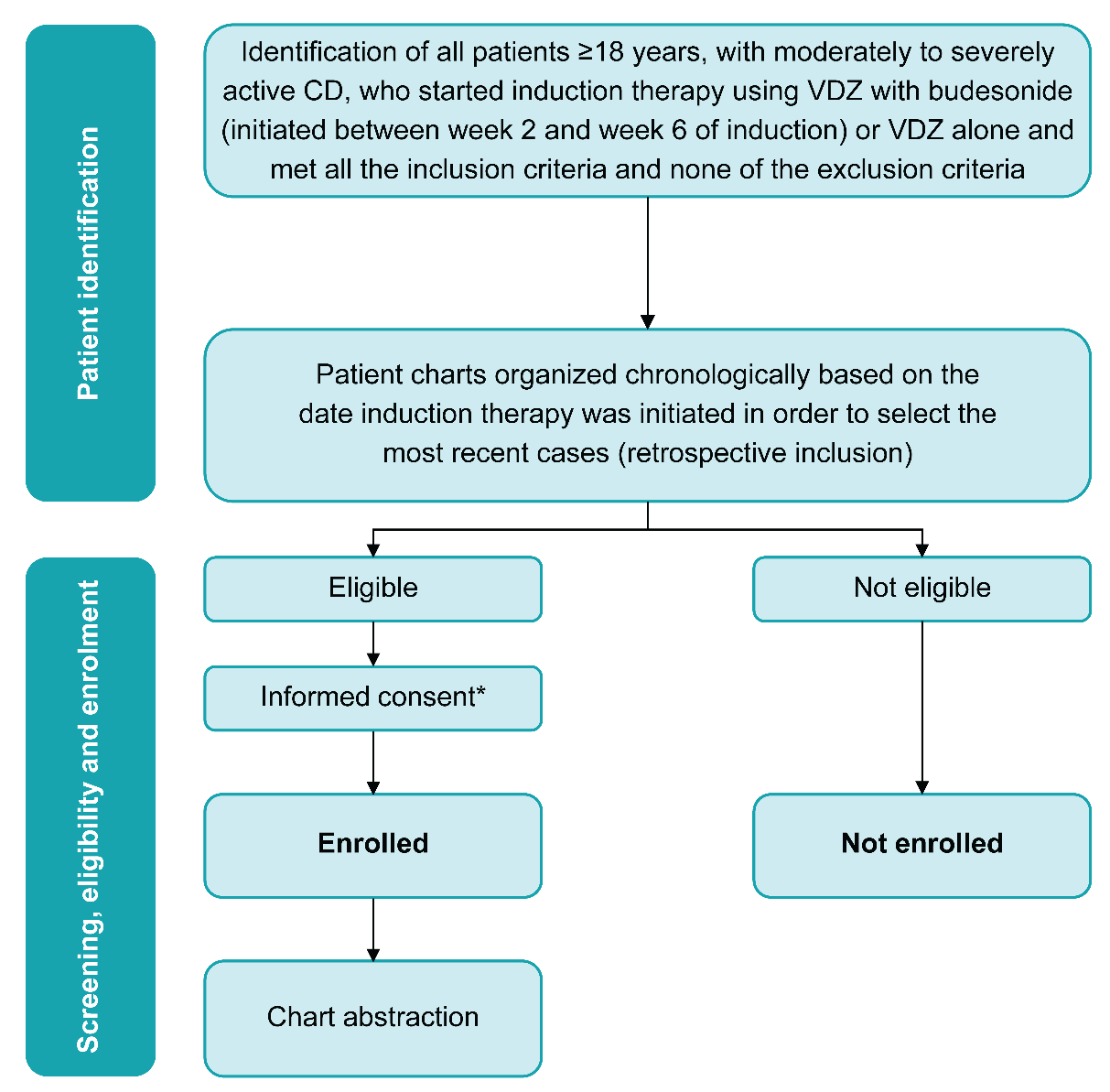


*Informed consent was collected, as appropriate, following local regulation

BUD = budesonide; CD = Crohn’s disease; VDZ = vedolizumab
