# Supplementary material for: Effectiveness and safety of vedolizumab induction with or without budesonide in patients with moderately to severely active Crohn’s disease in Europe: a retrospective observational study
Source: BMC Gastroenterol. 2023 Nov 29;23:417. doi: 10.1186/s12876-023-03032-7 (PMC10688148; doi:10.1186/s12876-023-03032-7)
Supplement: Supplementary file 2 — Supplementary Material 2 [file 12876_2023_3032_MOESM2_ESM.docx]

**Additional file 2.**

Supplementary Table 1: Demographic and disease characteristics at index date

| **Characteristic** | **VDZ alone (N = 73)** | **VDZ+BUD (N = 50)** | **Overall population (N = 123)** | **p-value** |
| --- | --- | --- | --- | --- |
| **Disease behavior (Montreal classification), n (%)** | | | | NA |
| **B1 non-stricturing,  non-penetrating** | 42 (67.7) | 28 (63.6) | 70 (66.0) |  |
| **B2 stricturing** | 10 (16.1) | 11 (25.0) | 21 (19.8) |  |
| **B3 penetrating** | 6 (9.7) | 0 (0.0) | 6 (5.7) |  |
| **B1 non-stricturing,**  **non-penetrating+perianal disease** | 1 (1.6) | 2 (4.5) | 3 (2.8) |  |
| **B2 stricturing+perianal disease** | 2 (3.2) | 1 (2.3) | 3 (2.8) |  |
| **B3 penetrating+perianal disease modifier** | 1 (1.6) | 2 (4.5) | 3 (2.8) |  |
| **Location of lesions^a^, n (%)** | | | | NA |
| **Rectum** | 5 (20.0) | 6 (35.3) | 11 (26.2) |  |
| **Sigmoid and left colon** | 6 (24.0) | 6 (35.3) | 12 (28.6) |  |
| **Transverse colon** | 3 (12.0) | 5 (29.4) | 8 (19.0) |  |
| **Ileum** | 22 (88.0) | 14 (82.4) | 36 (85.7) |  |
| **Right colon** | 6 (24.0) | 8 (47.1) | 14 (33.3) |  |
| **Type of lesions^a^, n (%)** | | | | NA |
| **Presence of deep ulceration** | 11 (44.0) | 9 (52.9) | 20 (47.6) |  |
| **Presence of superficial ulceration** | 22 (88.0) | 15 (88.2) | 37 (88.1) |  |
| **Presence of narrowing** | 1 (4.0) | 1 (5.9) | 2 (4.8) |  |
| **Presence of inflammation without ulceration** | 8 (32.0) | 4 (23.5) | 12 (28.6) |  |
| **Prior surgery for CD^b^, n (%)** | | | | 0.4664 |
| **Ileocolonic resection with ileocolonic anastomosis** | 24 (64.9) | 14 (63.6) | 38 (64.4) |  |
| **Ileocolonic resection with ileorectal anastomosis** | 2 (5.4) | 1 (4.5) | 3 (5.1) |  |
| **Partial colectomy** | 2 (5.4) | 2 (9.1) | 4 (6.8) |  |
| **Partial small bowel resection** | 4 (10.8) | 2 (9.1) | 6 (10.2) |  |
| **Stricturoplasty** | 1 (2.7) | 0 (0.0) | 1 (1.7) |  |
| **Perianal fistula surgery** | 2 (5.4) | 5 (22.7) | 7 (11.9) |  |
| **Chronic comorbidities, n (%)** | | | | NA |
| **Alzheimer's or severe**  **chronic cognitive**  **deficiency** | 1 (3.0) | 0 (0.0) | 1 (1.9) |  |
| **Asthma** | 1 (3.0) | 2 (9.5) | 3 (5.6) |  |
| **Cerebrovascular disease** | 3 (9.1) | 1 (4.8) | 4 (7.4) |  |
| **Chronic liver disease** | 2 (6.1) | 1 (4.8) | 3 (5.6) |  |
| **Chronic lung disease** | 2 (6.1) | 1 (4.8) | 3 (5.6) |  |
| **Congestive heart failure** | 3 (9.1) | 1 (4.8) | 4 (7.4) |  |
| **Connective tissue disease** | 1 (3.0) | 1 (4.8) | 2 (3.7) |  |
| **Depression** | 3 (9.1) | 4 (19.0) | 7 (13.0) |  |
| **Renal disease** | 3 (9.1) | 2 (9.5) | 5 (9.3) |  |
| **Diabetes** | 4 (12.1) | 1 (4.8) | 5 (9.3) |  |
| **Rheumatic disease** | 5 (15.2) | 5 (23.8) | 10 (18.5) |  |
| **Diabetes with end organ damage** | 0 (0.0) | 1 (4.8) | 1 (1.9) |  |
| **Solid tumor** | 3 (9.1) | 1 (4.8) | 4 (7.4) |  |
| **Eye disorder** | 3 (9.1) | 1 (4.8) | 4 (7.4) |  |
| **Transient ischemic disease** | 1 (3.0) | 0 (0.0) | 1 (1.9) |  |
| **Any other psychiatric disorder** | 2 (6.1) | 0 (0.0) | 2 (3.7) |  |
| **Any other skin disorder** | 3 (9.1) | 3 (14.3) | 6 (11.1) |  |
| **Hypertension** | 9 (27.3) | 3 (14.3) | 12 (22.2) |  |
| **Hypo/Hyperthyroidism** | 4 (12.1) | 1 (4.8) | 5 (9.3) |  |
| **Myocardial infarction** | 0 (0.0) | 2 (9.5) | 2 (3.7) |  |
| **Gastric or peptic ulcers** | 5 (15.2) | 3 (14.3) | 8 (14.8) |  |
| **Other^c^** | 13 (39.4) | 13 (61.9) | 26 (48.1) |  |

^a^Percentages calculated over the number of patients with lesions at index date (n = 42)

^b^Assessed since CD diagnosis until 12 months before the index date; percentages calculated over the number of patients with prior surgery (n = 59)

^c^Other comorbidities in patients on VDZ alone: anal fissure, five patients with iron deficiency anemia, hypovitaminosis D, two patients with vitamin B12 deficiency, osteopenia, IgG gammopathy, hypergammaglobulinemia benign monoclonal, ulnar nerve palsy, malignant melanoma of skin of lower limb (including hip), prostatitis, two patients with obesity, osteoporosis and atrial fibrillation

Other comorbidities in patients who received VDZ with BUD: chronic fatigue syndrome, coronary stent placement, two patients with bacterial overgrowth, radiculitis lumbosacral, uric acid nephrolithiasis, three patients with iron deficiency anemia, vitamin B12 deficiency, stress incontinence, irritable bowel syndrome, recurrence urinary tract infection, migraine, two patients with osteoporosis, myelitis transverse and pelvic floor dyssynergia

BUD = budesonide; CD = Crohn’s disease; IgG = immunoglobulin G; NA = not available; VDZ = vedolizumab
