# Supplementary material for: Effectiveness and safety of vedolizumab induction with or without budesonide in patients with moderately to severely active Crohn’s disease in Europe: a retrospective observational study
Source: BMC Gastroenterol. 2023 Nov 29;23:417. doi: 10.1186/s12876-023-03032-7 (PMC10688148; doi:10.1186/s12876-023-03032-7)
Supplement: Supplementary file 3 — Supplementary Material 3 [file 12876_2023_3032_MOESM3_ESM.docx]

**Additional file 3**

**Supplementary Table 2.** Description of budesonide treatments received at index date and during follow-up in the VDZ+BUD group

|  |  |  | **VDZ+BUD**  **(n = 50)** |
| --- | --- | --- | --- |
| **Daily dose of budesonide (mg)** | Mean (SD) |  | 8.5 (1.4) |
|  | Median [Q1, Q3] |  | 9.0 [9.0, 9.0] |
|  | (Range) |  | (3.0, 9.0) |
| **Changes in budesonide treatment regimen during follow‑up** | No change |  | 20 (40.0%) |
|  | Change^a^ | Dose reduction | 23 (76.7%) |
|  |  | Reduction in frequency | 7 (23.3%) |
|  | Reason for change in BUD treatment regimen | Classical tapering schedule for budesonide | 30 (100.0%) |
|  |  | Lack of effectiveness in the management of CD | 2 (6.7%) |
|  |  | Partial treatment response to the management of CD | 0 (0.0%) |
|  |  | Good effectiveness in treating CD | 0 (0.0%) |
|  |  | Adverse event | 0 (0.0%) |
|  |  | Tolerability | 1 (3.3%) |
|  |  | Unknown | 0 (0.0%) |
|  |  | Other | 0 (0.0%) |
| **BUD discontinuation** | No |  | 16 (32.0%) |
|  | Yes |  | 34 (68.0%) |
|  | Missing |  | 0 (0.0%) |
| **Reason for discontinuation** | Classical tapering schedule for budesonide |  | 29 (85.3%) |
|  | Lack of effectiveness in the management of CD |  | 2 (5.9%) |
|  | Adverse event |  | 1 (2.9%) |
|  | Good effectiveness in treating CD |  | 0 (0.0%) |
|  | Tolerability |  | 0 (0.0%) |
|  | Patient/family decision |  | 1 (2.9%) |
|  | Other |  | 1 (2.9%) |

^a^Percentage calculated over the number of patients with changes in budesonide treatment regimen during follow-up

All data are represented as n (%) unless otherwise stated

BUD = budesonide; CD = Crohn’s disease; Q = quartile; SD = standard deviation; VDZ = vedolizumab
